# Study protocol of a telephone problem-solving intervention for Spanish-speaking caregivers of veterans post-stroke: an 8-session investigator-blinded, two-arm parallel (intervention vs usual care), randomized clinical trial

**DOI:** 10.1186/s12875-022-01929-y

**Published:** 2023-03-17

**Authors:** I. Magaly Freytes, Magda K. Schmitzberger, Naiomi Rivera-Rivera, Janet Lopez, Keryl Motta-Valencia, Samuel S. Wu, Tatiana Orozco, Jennifer Hale-Gallardo, Nathaniel Eliazar-Macke, Jennifer H. LeLaurin, Constance R. Uphold

**Affiliations:** 1 Research Service, North Florida/South Georgia Veterans Health System, 1601 SW Archer Rd #151B, Gainesville, FL 32608 USA; 2 Geriatric Research Education and Clinical Center, North Florida/South Georgia Veterans Health System, 1601 SW Archer Rd #151B, Gainesville, FL 32608 USA; 3 Research Service, VA Caribbean Healthcare System, 10 Casia St, San Juan, PR 00921 USA; 4 Physical Medicine and Rehabilitation Service, VA Caribbean Healthcare System, 10 Casia St, San Juan, PR 00921 USA; 5 College of Medicine, Department of Biostatistics, University of Florida, CTRB Room 5243, 2004 Mowry Road, Gainesville, FL 32610 USA; 6 College of Medicine, Department of Aging and Geriatric Research, University of Florida, 2004 Mowry Rd, Gainesville, FL 32603 USA

**Keywords:** Hispanic Americans, Veterans, Caregiver Burden, Stroke Caregiving, Problem-solving, Depression, Culturally-relevant Intervention, Health Education, Quality of Life, Minority Health

## Abstract

**Background:**

Stroke is one of the leading causes of death and the main cause of long-term disability in the United States. The significant risk factors of stroke among Hispanics are well-documented. The majority of stroke survivors return home following a stroke and are cared for by family caregivers. Due to the abrupt nature of strokes, caregivers experience unexpected changes and demands that oftentimes lead to caregiver burden and depression. Given the significant risk factors for stroke in Hispanics and the influence of culture in family norms and family management, we developed a telephone and online problem-solving intervention for Spanish-speaking stroke caregivers. This study tests the impact of a telephone and online problem-solving intervention for Spanish-speaking stroke caregivers on caregiver outcomes.

**Methods:**

The design is a two-arm parallel randomized clinical trial with repeated measures. We will enroll 290 caregivers from 3 Veterans Affairs (VA) medical centers. Participants randomized into the intervention arm receive a problem-solving intervention that uses telephone and online education and care management tools on the previously developed and nationally available RESCUE en Español Caregiver website. In the usual care group, participants receive the information and/or support caregivers of veterans with stroke normally receive through existing VA resources (e.g., stroke-related information and support). The primary outcome is change in caregiver’s depressive symptoms at 1- and 12-weeks post-intervention. Secondary outcomes include changes in stroke caregivers’ burden, self-efficacy, problem-solving, and health-related quality of life (HRQOL) and veterans’ functional abilities. We will also determine the budgetary impact, the acceptability of the intervention and participation barriers and facilitators for Spanish-speaking stroke caregivers.

**Discussion:**

This is an ongoing study. It is the first known randomized controlled trial testing the effect of a telephone and online problem-solving intervention in Spanish for caregivers of veterans post-stroke. If successful, findings will support an evidence-based model that can be transported into clinical practice to improve the quality of caregiving post-stroke.

**Trial registration:**

ClinicalTrials.gov: NCT03142841— Spanish Intervention for Caregivers of Veterans with Stroke (RESCUE Español). Registered on February 23, 2018. Protocol version 8. 08.11.2022.

## Background

Stroke is one of the leading causes of death and the main cause of long-term disability in the United States (U.S.) [1]. By 2030 there is a projected 20.5% increase in stroke prevalence compared to 2012; the largest increase (29%) is projected amongst Hispanic men [2]. Cardiovascular disease is a leading cause of mortality among Hispanics in the US, and Puerto Ricans have the highest hypertension-related death rate among all Hispanics [3, 4]. A recent report from the American Heart Association and the American Stroke Association identified important issues that require the attention of researchers, such as culture and language barriers, accessibility issues, and bias among healthcare providers [5]. The economic burden of stroke on the healthcare system is also significant. The projected total cost of stroke from 2005 to 2050 (in 2005 dollars) for Hispanic individuals is $313 billion [2].

Due to the residual, and oftentimes debilitating, deficits of stroke, family caregivers become a major source of support for stroke survivors. Between 25 and 74% of stroke survivors have residual deficits and require some assistance, whereas other survivors are completely dependent on their caregivers to meet their daily living needs [6]. These unexpected changes experienced by family caregivers oftentimes result in high rates of caregiver depression [7, 8] and burden [9]. Studies on stroke caregivers report rates of depressive symptoms in 11% to 42% of this population [8] and caregiving burden has been found to be a main reason for institutionalizing stroke survivors [10]. Consequently, national practice guidelines [11, 12] underscore the importance of educating and supporting stroke survivors, and the family caregivers who manage their care.

Education and support programs for family caregivers have proven significant in facilitating the transition home following a stroke. Providing caregivers with information, support, and skills has the potential to reduce negative caregiver outcomes and increase the likelihood that stroke survivors can remain at home. Even though there is variation in the type and amount of information and support that caregivers need [13], multiple researchers found that individualized, tailored messages and support programs are more likely to improve caregiver outcomes than generic programs [14–18]. Offering education and support appropriate to the caregivers’ culture and preferred language is one way to tailor strategies and interventions for stroke caregivers. Further, interventions that include building skills to solve self-identified problems are more beneficial than other interventions [19, 20].

Caregiver studies examining face-to-face sessions indicate that these individual or group sessions were burdensome to caregivers who had limited time and energy. Moreover, they were found to be costly and labor-intensive [20]. While some studies evaluating the effectiveness of telephone problem-solving interventions report promising findings [21, 22], none have focused on Spanish-speaking caregivers. Therefore, it is uncertain if these telephone interventions would be equally effective within the Hispanic population.

The Internet is increasingly recognized as a cost-effective medium for disseminating up-to-date health information to large numbers of healthcare consumers. Research has shown that over 78% of Americans regularly use the Internet to obtain health information [23]. Adults 55 years and older (typical age range of stroke caregivers) are more likely to seek information on the Internet than adults in other age groups [24]. Over the last several years, the gap in Internet use between Hispanics and other groups has shrunk considerably, with Hispanics showing the highest increase in Internet usage (54% to 64%) [25]. The advantage of the Internet is that adults can receive health information at a convenient place and time.

Another gap in the stroke caregiving literature is that only a handful of studies have evaluated interventions and programs aimed at improving the lives of caregivers of veterans [26]. This is important because there are differences between veteran and non-veteran caregivers [26]. While caregivers of veterans face similar challenges as non-veteran caregivers, they also must contend with the issues related to combat-related injuries or disability, posttraumatic stress disorder (PTSD), depression, and greater health risks than the general population. Veteran care recipients also usually have lower incomes, higher rates of chronic disease, more comorbid conditions, and poorer health overall than their non-veteran counterparts [7].

Little is known about caregivers from cultural or minority groups, such as Hispanic caregivers of veterans. Studies show that in the general population, Hispanic caregivers exhibit more depression than white caregivers [27]. Other studies demonstrate that Hispanic dementia caregivers, who share similar challenges to stroke caregivers, have also been shown to have higher rates of depression compared to their non-Hispanic counterparts [28]. Given the importance of culture in affecting family norms and family management [29–32], it is critical that we expand our understanding of ways to enhance the important role that informal Hispanic caregivers play in the recovery and use of healthcare services by veterans post-stroke.

This study addresses an important and understudied area of caregiving research, Hispanic, Spanish-speaking caregivers of veterans who have suffered a stroke. We will focus on a Hispanic veteran population that geographically has been shown to have a high rate of strokes, Puerto Rican veterans. This study recognizes the important role that socio-cultural factors play in enhancing the skills of caregivers of stroke patients and addresses the need for culturally relevant caregiver programs. Although Hispanics are the fastest growing group in the military and the VA, there has been insufficient effort to reduce the health disparities in this population. All veterans are required to understand English, but many Hispanic family caregivers lack basic English-language skills and Spanish is their preferred language.

## Methods/design

### Advisory panel

We established an advisory panel consisting of clinicians and VA leadership at the national and local level who are independent from the funding agency to obtain input in the planning and development phases of the study. The study team and the advisory panel meet quarterly to discuss progress and challenges. Once data collection is completed, members of the advisory panel will assist in the interpretation of the study findings and planning for the dissemination of results and strategies to sustain the intervention in practice if it is found to be successful. Upon approval and funding, this study was not assigned a data monitoring committee, thus, our independent advisory panel serves as our monitoring committee.

### Objective and hypothesis

The objective of this study is to evaluate the impact of a telephone and online, problem-solving intervention on caregiver outcomes. Our primary aim is to reduce caregiver depression. Secondary aims are to: reduce caregiver burden, improve caregivers’ problem-solving abilities, self-efficacy, and quality of life, improve veterans’ functional abilities and determine the intervention’s impact on veterans’ healthcare utilization. Additionally, we will determine the intervention’s budgetary impact, and caregivers’ perceptions of the intervention. The primary hypothesis is that stroke caregivers in the intervention group will have fewer depressive symptoms compared to those in the usual care group. The secondary hypotheses are that caregivers of stroke survivors in the intervention will have less burden, better problem-solving abilities, higher self-efficacy, and higher quality of life compared to caregivers assigned to usual care and veterans will have better functional abilities and lower healthcare utilization.

### Study design

This is a blinded, two-arm parallel (intervention vs usual care), randomized clinical trial with repeated measures. The trial started recruitment in March 2018 and is still actively recruiting participants. The original protocol was to recruit study participants from the VA Caribbean Healthcare System (VACHS) in San Juan, PR and all study procedures were to be conducted over the phone from either the North/Florida South Georgia Veterans Healthcare System (NF/SG VHS) in Gainesville, FL or the VACHS in San Juan. Within a year from the initiation of the study, we submitted and obtain approval for a project modification to add two additional sites with the sole purpose of identifying potential study participants. Data collection is currently on going in Puerto Rico and Gainesville, Florida, USA.

### Study setting

**Fig. 1:**
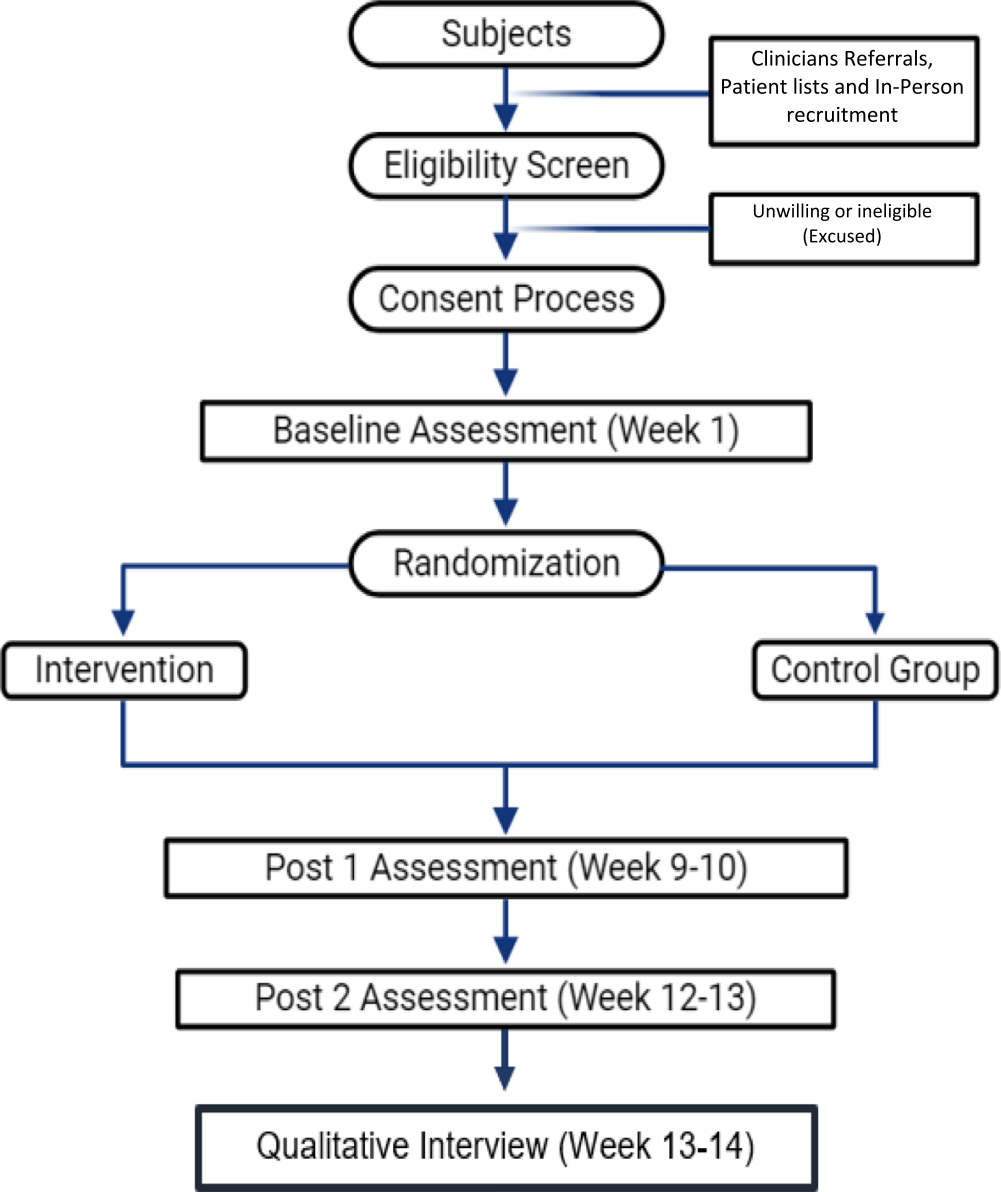
Flow chart of the study design

The main study site is the North Florida/South Georgia Veterans Health System which coordinates all study activities and lead all research procedures. Originally, the secondary site was the VA Caribbean Healthcare System, which continues to be the main recruitment site. Due to slow recruitment as a result of the impact of hurricane Maria in PR and in an effort to capture Puerto Rican families that migrated to the mainland, we added two additional VA, the Orlando VA Medical Center and the James A. Haley Veterans Hospital. All other study procedures (recruitment, data collection, etc.) are conducted from the North Florida/South Georgia Veterans Health System, in Gainesville, FL or the VA Caribbean Healthcare System, in San Juan, PR. A flow chart of the study design is shown below in Fig. 1.

### Eligibility criteria

All Hispanic caregivers of veterans with a primary diagnosis of stroke are eligible for participation if they meet the following criteria: 1) are the primary caregiver and provide the majority of care for a veteran who has a diagnosis of stroke (ICD9 codes for stroke: 430–438 or ICD 10 codes 160.0 through 169.998) within the last year and who have at least two activity of daily living (ADL) deficits or a new or worsening neurological problem as measured by a score of 74 or lower in the Stroke Impact Scale (SIS-16), 2) have Internet access and ability, (either themselves or via a relative or friend) 3) are reachable by cell or home phone, 4) Spanish is their preferred language, 5) have moderate to severe stress as measured by a score of 1 or higher on the Perceived Stress Scale (PSS-10), 6) self-identify as Hispanic, and 7) agree to random assignment to the intervention or usual care group. We determine caregiver status using previous research guidelines that indicate that one individual is typically identified as the primary caregiver and is responsible for the majority of caregiving tasks. We determine time since stroke by reviewing the veterans’ electronic health record (EHR). Deficits in activities of daily living [ADL] are assessed by asking caregivers if the Veteran is unable to complete any of the ADLs on the Stroke Impact Scale 16 (SIS-16). Caregivers of Veterans who had either embolic or thrombotic strokes are included because there are no previous studies that indicate a differential impact of stroke type on caregiver outcomes. We do not limit the study to caregivers of first-time stroke survivors. Hispanic/Hispanic ethnicity and Internet access and ability to use are determined by caregiver self-report. We exclude caregivers who are managing end-of-life issues (stroke survivors who are likely to die within five months following discharge). Life expectancy is determined by reviewing the EHRs and conferring with our physician team members.

Since the intervention is conducted over the phone and to reduce participation burden, we obtained a Waiver Documentation of Informed Consent. However, members of the research team trained in informed consent procedures utilize an Informed Consent Written Statement to inform interested and eligible participants about the purpose of the study, study procedures including that some sessions may be recorded, risks, volunteer nature of their participation, potential benefits, and who to contact in case of questions or concerns, etc. The written statement is also used to inform and obtain oral permission to record the interventions. A copy of the written statement is mailed to participants prior to reviewing the Informed Consent Written Statement. All informed consent procedures are conducted over the telephone by trained and experienced members of the research team from either the NF/SG VHS or the VACHS. To collect veteran healthcare utilization data, we ask caregivers to self-report.

### Intervention

The intervention is based on the relational/problem-solving model of stress.

developed by D-Zurilla and Nezu [33] and integrates concepts from Lazarus’ stress appraisal and coping theory [34]. The model can be summarized by the acronym COPE (Creativity, Optimism, Planning, and Expert Information) [35] and has been translated into Spanish.

Session 1. Tour of the Spanish RESCUE Website and orientation to the problem-solving approach. During this session the interventionist builds rapport with the study participant, discusses the purpose, goals and scope of the intervention, and discusses his/her role in the intervention and the expectations for study participants. The interventionist reviews the content in the RESCUE workbook that the caregivers receive in the mail and ensures that the caregiver can access the RESCUE website. The interventionist then gives a tour of the various features on the RESCUE website as the participant simultaneously navigates and explores the website. Next, the interventionist directs the participant to the problem-solving module (either online or in the workbook) to teach the COPE problem-solving approach which involves the following steps: 1) identifying & defining problems; 2) prioritizing problems; 3) selecting the highest priority problem; 4) gathering expert information; 5) setting realistic goals; 6) listing all possible solutions; 7) choosing the best solution; and 8) evaluating the plan. The interventionist assesses participant’s understanding before discussing an illustrative example on how to apply the problem-solving approach to caregiver problems, and to use of the problem-solving diary and the materials in the RESCUE website. Session one concludes with a summary of caregiver stress and depression, assignment on the next topics for discussion, and scheduling session two with the caregiver.

Session 2. The interventionist and study participant discusses assigned topics and use the problem-solving diary and RESCUE website to work through the caregiver problems. The interventionist begins the session assessing any changes in the caregiver and veteran. Next, the interventionist discusses the content on the pre-assigned topics and addresses any questions. The study participant and the interventionist then collaborate to develop a personalized problem-solving plan to address participant’s caregiving problems using the problem-solving diary which can be accessed and printed from the RESCUE website. The interventionist uses motivational interviewing [36] and encourages the participant to talk about their caregiver challenges. The interventionist can also review the list of topics/factsheets that are on the RESCUE website to trigger identification of problems. The study participant uses the diary to write down and prioritize their caregiving problems, identifying the most troublesome. The interventionist then empowers the participant to identify realistic goal(s), possible solutions, best solution, and evaluation plans, which are recorded in the problem-solving diary. At the conclusion of session two, the interventionist addresses any questions and highlights the major points of discussion. The next session is also scheduled and three fact sheets from the RESCUE website are assigned for the following session.

Sessions 3–7. The interventionist and study participant continue to discuss assigned topics and use the problem-solving diary and RESCUE website to work through the caregiver problems. These sessions are very similar to the previous one. The assigned readings for sessions 4—7, are chosen based on the caregiver problems and priorities identified in the previous session. In session seven, the interventionist also alerts the participant that the next session will be their last.

Session 8. Summary of the problem-solving approach. The interventionist begins by assessing any changes in the participant and veteran. Next, they review and discuss the assigned topics for the session. The interventionist then works with the study participant to review and finalize the personalized problem-solving plan using the problem-solving diary and, identify information related to newly identified caregiver problems on the RESCUE website. The session ends with a summary of what they learned throughout the intervention and motivational message to empower the participant to continue using the RESCUE website and to regularly evaluate their personalized problem-solving plan.

### Usual care

To reflect the current real-world experience of caregivers, we selected usual care as the control condition in this study to determine if the intervention is superior to usual care. Participants in the standard care group receive the care that is normally provided to stroke caregivers of Veterans. Caregivers do not normally receive any follow-up telephone or transitional care unless they are enrolled in the telehealth or home-based primary care programs (study exclusionary criteria). Stroke caregivers may only receive contact information for stroke clinicians, and some may receive informational brochures or an invitation to participate in an open support group led by the psychologist in the rehabilitation clinic. At the end of the study, we will ask caregivers in the standard care group about resources used throughout the study.

### Fidelity considerations

We make special effort to enhance, maintain and track treatment fidelity. Study personnel use a study manual that provides step-by-step guidelines for recruitment, enrollment, informed consent, intervention and data collection procedures. All members of the study team receive extensive training on all pertinent study procedures. In addition, ten percent of the intervention sessions are observed either in-person or from a digital recording by the study PI using a fidelity checklist developed by the study team to measure adherence to the protocol, therapeutic communication skills, delivery of intervention content, integration of the COPE model, decision-making skills, and response to caregiver problems. This tool is used to document deviation from study protocols and give feedback to the interventionists to ensure fidelity.

To improve subject adherence, study staff provide reminder phone calls one day prior to the weekly session. The interventionists also emphasize the importance of following study procedures during the first intervention session and have staggered schedules to offer participants a wide variety of availability including early morning and early evening.

At the final session we make sure there is a smooth transition and hand-off by re-iterating where the study participants can find additional resources once the intervention is over. This includes making sure they have contact information for their VA primary care provider, social worker, and the VA Caregiver Support Line. We also remind them about the online availability of the entire RESCUE website and we give participants in the usual care group the address for the RESCUE en Español website.

Participants who are unable to be contacted or unable to adhere to the intervention schedule are administratively withdrawn. Participants whose care recipient dies or are institutionalized are withdrawn. If the subject experiences worsening or severe stress or burden that prevents them from fully participating in the study, we re-assess their current situation and if it is determined to be in their best interest, they are withdrawn.

## Measures

The primary outcome is caregiver’s depressive symptoms. Secondary caregiver outcomes are stroke caregivers’ burden, self-efficacy, problem-solving, and health-related quality of life (HRQOL). Secondary stroke survivor outcomes are change in functional abilities. All outcomes are measures three times, pre-intervention (week 1 for participants assigned to the usual care group) and at 1- and 12-weeks post-intervention (9 and 21 weeks after baseline for participants assigned to the usual care group).

Depressive symptoms is assessed using the Center for Epidemiologic Studies Depression [37] (CES-D 20, Spanish Version) The CES-D is a 20-item, 4-point Likert scale ranging from never (0) to most of the time (3). It assesses depressive symptoms and is not a clinical measure of depression [37]. The total score of the CES-D 20 is obtained by adding all the items. Scores range from 0–60 with higher scores indicating greater depressive symptoms. If more than 4 items are missing, the CES-D is not scored. This tool has been used in numerous studies with caregivers and has good reliability and validity [38].

Caregiver burden is assessed using the Zarit Burden Interview (S-ZBI, Spanish Version)[39]. This is a 22-item instrument with good validity and reliability. The ZBI uses Likert items on a 5-point scale to measure the degree of burden felt by caregivers. Items fall into five categories (health, well-being, finances, social life, relationship with impaired person). The total score of the ZBI is obtained by adding all the items. High scores indicate greater burden. We selected this tool above other burden instruments because it is brief, easy-to-answer, measures both subjective and objective burden, and is a widely-used [39].

Self-efficacy is assessed using the Caregiver Self-Efficacy (Spanish Version) [40]. This is a 15-item measure of caregivers’ judgments regarding their ability to perform effectively in specific, caregiving settings. The measure is composed of three domains: obtaining respite, responding to disruptive patient behaviors, and controlling upsetting thoughts. Respondents rate their level of confidence for each item on a scale from 0 to 100, with higher scores corresponding to greater confidence. The item scores within each domain are averaged to obtain subscale ratings ranging between 0 and 100. The tool has established reliability and validity. We selected this self-efficacy tool because it was developed specifically for caregivers and has two subscales that are related to our problem-solving framework.

Problem-solving abilities are assessed using the Social Problem-Solving Inventory Revised—Short Form (SPSI-R:SF) [41] (Spanish Version). The SPSI-R-SF is a 25-item tool consisting of five subscales (positive problem-solving orientation, rational problem-solving orientation, negative problem-solving orientation, impulsivity or carelessness style problem solving, avoidance style problem solving). Items on the SPSI-R are rated on a 5-point Likert-type scale (0 = not at all true of me to 4 = extremely true of me) with higher scores on each factor indicating greater intensity on a particular dimension. Each subscale is scored individually by summing the items and converting to age-normed scores, and a total standardized score is obtained by reverse-scoring the appropriate subdomains (NPO, ICS, and ACS), summing the subscale scores, and converting to an age-normed total score. Thus, both total score and separate domain scores can be obtained. We chose this tool because it has been used in previous, stroke caregiver studies, it has good psychometric properties, and it is briefer than the original 52-item tool [42].

Quality of life is assessed using the VR-12 RAND 12-Item Health Survey [43] (Spanish Version). The VR-12 is a multi-use, health survey comprised of 12 items. The instrument is primarily used to measure health-related quality of life, to estimate disease burden and to evaluate disease-specific benchmarks with other populations. The VR-12 items are scored on a 3-point, 5-point or 6-point Likert scale consisting of two scales: “Physical Health Summary Measure” and “Mental Health Summary” Measure”. Scale scores are obtained by running the scoring algorithm provided by Kazis et al. [44] The VR-12 has good psychometric properties [45].

Veterans’ function ability is assessed using the Stroke Impact Scale-16 [45] (Spanish Version), It is comprised of 16 short questions on a 5-point scale and is used to assess physical function of patients with stroke. Appropriate items are reverse-scored, and the total score is transformed to be on a scale of 0–100, with higher scores corresponding to higher physical function abilities. The scale has good psychometric properties and has been used extensively in studies of outcomes of care in rehabilitation settings [46]. We screen caregivers by asking them if their stroke survivor needs assistance performing the tasks.

Veterans’ healthcare utilization data (i.e., unintended hospital bed days of care, number of emergency room visits, number of unscheduled clinic visits) during the study period time are obtained by caregivers’ self-report. We record the number, dates, and the reasons for all healthcare visits.

## Other measures

For caregivers we use the Problem Checklist [47], as a screening tool. This instrument developed via consensus by members of the research team with expertise in nursing, psychology, and public health based on published caregiving literature [48, 49] and items in the World Health Organization’s (WHO) International Classification of Functioning (ICF) [50]. This 20-item tool measures common caregiving problems (and stroke survivors’ problems and is only administered during the baseline data collection. Study participants respond “yes” or “no” to indicate whether the problem had occurred in the past month. This tool is used to obtain a better understanding about the challenges participants were experiencing at the beginning of the study. If later during the intervention participants have trouble identifying caregiving related problems, the interventionists use this data to help them generate ideas. We also use the Perceived Stress Scale – PSS10 (Spanish) [51] as a screening tool for caregiver. This is a 10-item 5-point Likert scale ranging from never (0) to very often (5) used to evaluates level of perceived stress. It is ideal to be used It has been used in numerous studies with caregivers and has good reliability and validity [51]. Both tools are used stress prior to study enrolment. The Stroke Impact Scale-16, one of the Veterans secondary outcomes, is also used as a screening tool for caregivers prior to enrolment to determine how much assistance stroke survivor needs to perform these tasks.

Budgetary impact. To determine the cost of the intervention itself, we will obtain Veterans’ health care utilization data at the end of the study. We will use the average elapsed time of intervention sessions along with an estimate of the average national wage of the type of interventionist most likely to deliver the intervention in the field. Data on VA-funded use costs will be obtained from Managerial Cost Accounting System (MCAS) and the Non-VA Medical Care files.

**Fig. 2:**
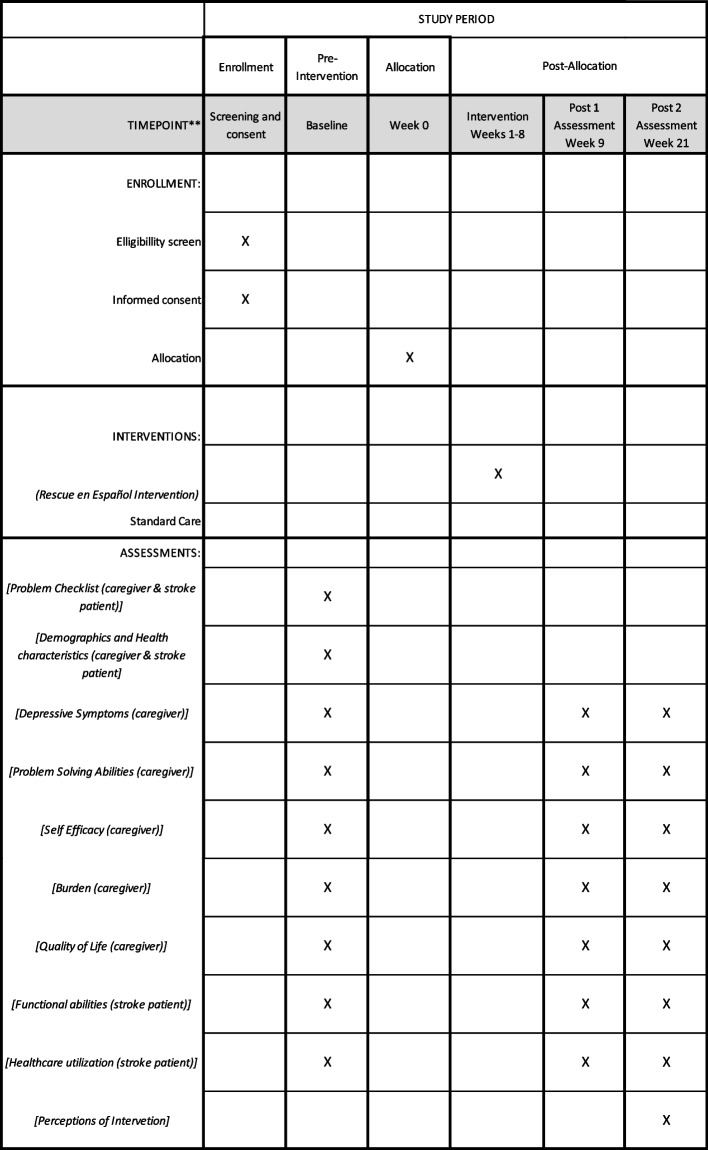
Schedule of enrollment, interventions, and assessments

Enactment Tool (Translated). We used experienced translators to adapt and translate the Bakas, et al.,[52] enactment tool. Translations were then verified by native Spanish-speaking members of the research team who met to discuss and finalize the Spanish adaptation. This tool evaluates treatment acceptability and enactment by asking caregivers who complete the intervention to rate the amount of information and contact they received during the 8-sesssion intervention, how much they used the problem-solving strategies and the RESCUE en Español website, how helpful the intervention was and whether their caregiver problems were resolved. It consists of 8 closed-ended questions with a 5-point scale and three open-ended questions for participants to elaborate on what they like best about the intervention and how it can be improved. Participant timeline including enrollment, interventions, and assessments is show in Fig. 2.

## Sample size

### Quantitative methods

The sample will consist of 290 caregivers. Pilot data from a previous study showed 3.1 points reduction in CES-D with a within group standard deviation of 8.9. Assuming such an intervention effect size on depression, 145 subjects per group would achieve 80% power to detect the difference at a 5% significance level. The sample size of 145 per group (intervention vs. standard care) was selected to account for the occurrence of a 10% dropout rate. The 10% drop-out rate is based on a one of a previous stroke caregivers study conducted by members of the team. The assumed effect size and variability were deemed reasonable based on a number of other sources. Radloff [37] described large changes pre and post treatment in a clinical setting, with depression from time of admission to 4 weeks post decreasing 20 points for “recovered” patients and 12 points for “still ill” patients. Grant et al. [22] in a study of a telephone intervention to family caregivers of stroke caregivers, found an estimated 1-month treatment effect of 3.1.

### Qualitative methods

Sample size considerations in qualitative studies include the purpose/goal of the study and the depth of data needed to reach theoretical saturation and adequately answer the study question [53]. Typically, 8–12 participants are needed to reach theoretical sample [54]. We will select a purposive subsample of 8–12 caregivers who participated in the intervention arm of the project. We will sequentially enroll every sixth caregiver who completes the intervention until we have the desired sample size or theoretical saturation.

Recruitment and enrollment procedures are guided by procedures that have been used successfully to enrol caregivers in our pilot project and other caregiver studies. VACHS recruitment site procedures: We have three methods for identifying potential study participants. 1) Patients List. Each month, we obtain a list of stoke patients with ICD9 codes (430.00—438.99) and/or ICD10 codes (160.0—169.98) from the Health Administration Service (HAS) to review patient EHR to develop a list of potential subjects. We have a full Health Insurance Portability and Accountability Act (HIPAA) Waiver for patient screening and record review to verify diagnosis and identify next of kin. We mail invitation letters explaining the purpose of the study to potential study participants (caregivers of veterans post-stroke) and contact number to call to decline to participate in the study. If we do not receive a response, we contact them in 7–10 days. 2) Clinicians Referrals. Clinicians will inform potential participants about the study and refer them to the study team if interested. 3) Referrals from Another Study. We also receive referrals from a similar study testing a similar English-language version of our intervention if potential participants prefer to receive the intervention in Spanish.

At the James A. Haley Veteran’s Hospital site we use clinician referral recruitment procedures (these recruitment procedures were approved by the University of South Florida IRB (IRB#: Pro00039920) and in the Orlando VAMC, we use patient list and clinician referral in our recruitment procedures (these recruitment procedures were approved by the Orlando VA IRB (Study #1,400,648–1). Recruitment calls to potential participants identified from any of the three sites through the procedures described above are conducted by trained, Spanish speaking study staff in either Gainesville or PR. In addition to explaining study purpose and procedures, during the recruitment phone calls we will screen potential participants for study eligibility. All study procedures will be conducted over the telephone.

### Randomization

The study statistician implements the Pocock-Simon covariate adaptive randomization procedure [55] so that an approximately equal number of caregivers are assigned to the two groups within each covariate level. To conceal the sequence until participants are assigned to the study arm, we use opaque, sealed envelopes. The envelopes are opened by an unblinded member of the study team who conducts the baseline data collection at completion of baseline assessment while participant is on the phone. The data collector notifies participants of their assignment and advises on next steps.

### Blinding

The data collector who conducts all the post-intervention assessments and the study PI will be blinded to the participants’ group assignments. The study database is secured with a two-way accessibility in which study team members who are not blinded have a password to access any information that may reveal participant’s assignment.

### Safety considerations

In the event of a safety concern (e.g., abuse, suicidality), the PI will be notified of the situation immediately after the safety concern arises to address the situation and determine participants continuation or need for withdrawal from the trial. In the event a data collector’s blinding was compromised for an individual participant they are not permitted to do future data collections for that participant. This can occur because of the format of the data collections (e.g., participant mentions doing intervention sessions).

### Data collection and management

#### Quantitative data

All data collectors in the study are experienced data collectors and were trained specifically on the data collection tools used in this study. Baseline data collection is scheduled shortly after informed consent procedures are completed over the telephone at a day and time convenient to the participant. Data collectors record participant responses on paper and then enter them into an online database and data quality checks are conducted and entered by staff. A study member who did not enter or collect the data checks paper data collection forms against database entries to assure that that information is accurate and no data are missing. The two data collectors do not know participants’ group assignment.

#### Qualitative data

In-depth qualitative interviews are conducted using a semi-structured interview guide consisting of 22 questions developed by the research team for this study. These in-depth interviews about participants’ perception of the intervention address three main topics: feedback about the components of the intervention, the usefulness of the intervention, and the study procedures. Interview questions ranged from asking participants to share their thoughts about the quantity and breadth of the stroke related topics covered during the intervention to what they would change about the intervention and what barriers, if any, they face throughout the study.

Qualitative interviews are conducted by experienced fluent Spanish-speaking interviewers using an interview script over the telephone. Interviewers receive training on the study interview scrip. Interviews are digitally recorded, transcribed verbatim, and validated by another Spanish-speaking member of the research team.

#### Participants retention

TO promote participant retention and completion of follow-up, we conduct reminder calls before sessions or data collections and have expanded scheduling hours to accommodate participants busy schedules. We do not collect any additional data on participants who withdraw from the study, but we do track reasons for withdrawing.

#### Confidentiality

We conduct intervention and data collection sessions in a private and secured area. We use encrypted voice recorders to record intervention sessions for fidelity checks and qualitative interviews. The audio sessions are downloaded and stored on a protected, secure VA research server, and then deleted from the portable device. Digital recordings use first names only and any identifying information is be removed during the transcription. All data files are kept in secured spaces and stored on a secure VA serve that has limited access to only study personnel.

### Data analysis

The primary analysis is to examine the effect of the intervention on caregiver depression based on “intention to treat.” Data from all the participants will be part of the primary analyses regardless of actual number of completed sessions. As an exploratory study element, we will assess compliance and attempt to determine its effect on study results. For the primary analysis, the general linear mixed model for repeated measures will be used to model the follow-up depression times (1- and 12-weeks post-intervention or 9 and 21 weeks for usual care), adjusted for baseline scores as fixed effects. Let yij be the depression score for person i at time j, then basic fixed effects model will be:

$$\mathrm E(\mathrm{yij})\:=\:\mathrm\beta0\:+\:\mathrm\beta1\mathrm{Baselinei}\:+\:\mathrm\beta2\mathrm{Timeij}\:+\:\mathrm\beta3\mathrm{Groupi}\:+\:\mathrm\beta4\mathrm{Groupi}\ast\mathrm{Timeij}$$

with covariates for baseline depression (Baseline), time (Time = 0 for 1 week, 1 for 12 weeks post-intervention or 9 and 21 weeks for usual care), and group (0 if active control, 1 if intervention). In order to control for possible chance sample imbalances resulting from randomization, the model will include covariates for baseline prognostic factors (e.g., caregivers’ relationship to veteran; number of previous strokes among veterans, location, etc.), deemed to have significant relationships with response and groupwise imbalances. Thus, analyses will be able to compare the groups on the measure of interest while controlling for these factors. The primary outcome of interest will be the immediate post-intervention effect at 1-week postintervention with the 12-week post-intervention as a secondary outcome. This will be tested based on linear contrast β3 + β4 at two-sided 0.05 level. All tests will be performed at the α = 0.05 significance level. The correlation among observations from the same person will be accounted for through the inclusion of a random person effect into the mixed model analysis. Data analysis for the secondary outcomes (i.e., burden, problem solving skills, self-efficacy, and HRQOL) will follow the plan for our primary outcome. No data analysis will take place until all data has been collected.

The analysis to determine the intervention’s budgetary impact consists of two parts: (1) the incremental cost of the intervention itself over and above usual care, and (2) the impact of the intervention on healthcare utilization. Micro-costing techniques [56] combined with average costing [57] will be used to determine the average staff time, wage, space, and equipment costs associated with the intervention. The micro-cost estimate for the telephone sessions will use the average elapsed time of such sessions along with an estimate of the average national wage of the type of nurse most likely to deliver the intervention in the field. To determine the intervention’s impact on the costs of healthcare utilization, the team will rely on the Professional Society for Health Economics and Outcomes Research ISPOR 2014 budgetary impact analysis guidelines [58]. Data on VA-funded utilization costs will be obtained from Managerial Cost Accounting System (MCAS) and the Non-VA Medical Care files. The team will tabulate all costs from these sources for study enrollees throughout the study, calculate the difference between intervention and usual care average costs, and test for the statistical significance of this difference using the Z-score method proposed by Zhou [59]. The final step in determining the budgetary impact of the intervention will combine parts 1 and 2 to determine the complete impact of the intervention on the VA budget.

The qualitative data analysis will consist semi-structure interviews to investigate the caregivers’ perceptions of barriers and facilitators and the acceptability of the intervention. We will invite every 6th study participant assigned to the intervention who completes the study to participate in a semi-structured interview. Once all the interviews have been conducted, study team members will begin the analysis by reading the first two caregiver transcripts and use template analysis to organize and analyse qualitative data using a priori themes based on the qualitative interview guide [60]. We will apply this framework to the first two interviews and add or revise themes as needed. Team members will independently code each transcript and l and meet to discuss coding decisions and resolve discrepancies. Data analysts will meet regularly with other team members to discuss the findings and search for alternative explanations in the data continuing this iterative process until no new themes are identified. To ensure rigor we keep an audit trail containing a log of all decisions and changes, along with the reason for the decision or change. Data will be analysed using NVIVO (QSR International) software.

Quantitative data obtained from the Enactment Tool (Bakas) [52] in which the caregiver rates aspects of the intervention, combined with qualitative data, will help us understand which aspects of the website and intervention were most beneficial and how the perceptions about the strengths and weaknesses of the various parts of the intervention varied between caregivers with different depression levels. We will use simple descriptive statistics to analyse quantitative data and thematic analysis for the qualitative data. Findings will provide data to help refine methods for future testing and roll-outs.

The measures for problem-solving and quality of life have methods for imputing missing scores on individual items. In the event of participant withdrawals, the missingness mechanism will be explored. If the assumption of Missing Completely at Random (MCAR) or the common assumption of Missing at Random (MAR) are plausible, then little change to the analysis plan is necessary. The mixed models analysis using direct-likelihood approaches will be unbiased and appropriate. Mixed effects modeling requires large samples but does not require full data on each subject.

Study efficiency (power) will of course be affected by the smaller amount of available information, thus we have inflated the planned recruitment sample size [61, 62]. It is important to note however, that it is rarely the case for missing data to be truly and purely MCAR; missingness may be multifaceted and portions of data may be missing at random vs missing systematically, in a way related to outcome of interest. Thus, even if missingness and attrition is not completely random (i.e., the cause of missingness or dropout is not related to the outcome of interest), missing data handling techniques will still be valuable, and we will employ one of a few options available. We may choose to use either multiple imputation (MI) or full-information maximum likelihood (FIML) estimation with the mixed effects regression models proposed. If there is a portion of missingness that is missing not at random, then parameter estimates may still be biased and should interpreted cautiously, but power will be less affected. Ultimately, even if there are data missing not entirely at random, these techniques are better alternatives than the default of listwise deletion of participants with missing data (which would introduce both bias and loss of power).

## Discussion

Stroke caregiving has been associated with higher rates of caregiver burden and depression [7,8]. Because majority of stroke survivors return home after a stroke, family members take on the roles of caregiver, oftentimes with little knowledge or skills to handle the sequalae of stroke survivors and the negative toll on their own physical and mental health. Thus, it is imperative to provide stroke caregivers with resources in order to adequately prepare them for their caregiver role and help alleviate the negative impact of caregiving.

The RESCUE intervention offers an alternative to traditional face to face interventions and if found to be successful, easily implemented in clinical setting. The ability to receive the intervention over the telephone minimizes caregiver burden and requires less healthcare system resources compared to in-person interventions. It is also especially relevant to the Hispanic population’s preferences for more direct contact with healthcare providers [63].

This is the first study that uses a culturally-adapted intervention specifically for Hispanic stroke caregiver. Unlike most intervention studies, this study includes an evaluation of the budgetary impact of the intervention and an advisory panel to collaborate throughout all the study phases and guide future plans for wider implementation. Once the study is completed, findings will be disseminated via professional publications and conferences, and reports to sponsor agency and programs. We will collaborate with stakeholders and members of our Advisory Panel to find additional venues for disseminating findings.

We have experienced some operational issues with this study. Lower recruitment than expected, due to natural disasters affecting our target population and negatively impacting the communication infrastructure in Puerto Rico, led to a revision in study procedures to add additional recruitment sites where many Puerto Ricans migrated to after these natural events. Issues with broad-band and telecommunications have also negatively impacted our ability to reach study participants in a timely manner.

## Conclusion

This study addresses an important and understudied area of caregiving research, Hispanic, Spanish-speaking caregivers of veterans who have suffered a stroke. This is the first known to evaluate a telephone and online problem-solving intervention combined with the nationally available Spanish- language, evidence-based RESCUE website. The intervention has the potential to reduce stroke caregiver depression, improve the recovery of veterans post-stroke, enable veterans to remain in their homes, and reduce healthcare costs. Another outcome will be a state-of-the art, nationally available culturally relevant website that both, family caregivers and health care providers can use for patient and caregiver education.

This trial is ongoing and is using protocol version #8 dated 08. 011.2022. Recruitment procedures started March 2018 and are expected to end in March 2023.

## Data Availability

Currently, only investigators associated with this study will have access to the final dataset. Final de-identified will be disclosed upon request per VA rules and regulations. Dissemination of findings and final study data will be available without restriction, in accordance with VHA directive 1200.19.

## Abbreviations

ADL: Activity of daily living
CES-D 20: Center for Epidemiologic Studies Depression
COPE: Creativity, Optimism, Planning, and Expert Information
EHR: Electronic Health Record
FIML: Full-information maximum likelihood
HAS: Health Administration Service
HRQOL: Health Related Quality of life
ICD: International Classification of Disease
ICF: International Classification of Functioning
ISPOR: Professional Society for Health Economics and Outcomes Research (formerly known as the International Society for Pharmaeconomics and Outcomes Research)
IRB: Institutional Review Board
MAR: Missing at Random
MCAR: Missing Completely at Random
MCAS: Managerial Cost Accounting System
MD: Medical Doctor
MI: Multiple Imputation
NF/SG VHS: North Florida/ South Georgia Veterans Healthcare System
PI: Principal Investigator
PSQ-III: Patient Satisfaction Questionnaire
PSS-10: Perceived Stress Scale
PTSD: Post-Traumatic stress disorder
R & D: Research and Development
RESCUE: Resources and Education for Stroke Caregivers’ Understanding and Empowerment
SIS-16: Stroke Impact Scale
SPOI-RSF: Social Problem-Solving Inventory Revised – Short Form
S-ZBI: Short Version of Zarit Burden Interview
US: United States
VA: Department of Veteran Affairs
VACHS: Veteran Affairs Caribbean Healthcare System
WHO: World Health Organization
